# The Week's Work

**Published:** 1910-09-10

**Authors:** 


### The Week's Work.

### THE RE-OPENING OF THE NEWCASTLE INFIRMARY CHAPEL.

The knowledge ordinary lay persons may have of the Newcastle-upon-Tyne Infirmary will probably in most cases be just a recollection that this was the institution benefiting, much to the disgust of that doughty henchman, under the will of the "fore-elder" of the celebrated Mr. James Pigg. Hospital workers, on the other hand, remember it as an exceptionally handsome building, recently rebuilt, supported to a large extent by colliery workmen, which during the last four or five years has notably increased its already large number of patients living outside the metropolis of the Northern Counties. Early in 1908 the Annual Court of Governors passed a resolution closing its chapel. This action was due to the friction consequent upon the following train of events. First, the chapel, apparently on the initiative of the House Committee, was consecrated under the auspices of the Church of England. Legal warrant thereby arose for its exclusive use by that religious body, members of which, by the way, had been prominent in contributing to the outfitting of the building. Facilities were provided for other denominational worship, but some of the Nonconformists showed themselves discontented. The upshot of the matter was as mentioned. Now, after two years and something, in consequence, it is to be gathered, of the decline of subscriptions because the chapel, erected from the funds of subscribers, was not opened to all Christian denominations on the same terms—after two years the resolution of 1908 is rescinded, and facilities will be given for "Anglican, Free Church, and such other services as may be required in the interests of the patients and staff."

### HOSPITAL CHAPELS.

Obviously the Newcastle Infirmary governors must be congratulated on the common-sense course they have taken. In India, we believe, the same trouble has occasionally arisen over use of the garrison churches provided for British troops, to be settled in the same way. And if conflicts between rival sects still occur at the Holy Sepulchre in Palestine, it is probably just because the Oriental is more excitable than the Anglo-Saxon, and the Turkish authorities less active or less sensible than the British ones. Since it is most unlikely that Church of England adherents will now outrage public opinion by going to law about the matter, only two other points appear to call for notice. One is the hyperbole of the Bishop of Newcastle's declaration that "England is looking on": the other, something more important, to wit the tone of the speech of Mr. Cairns, representative of the Northumberland Miners' Association, upon whose motion it was the chapel was shut up two years ago. This gentleman said he desired to point out that other folks contributed to the support of the institution besides Nonconformists and Churchmen; a great body of men, for instance, went to no place of worship at all, while others went to places other than those mentioned. The suggestion is distinct enough in these last remarks, of course, coming as they do on top of the past action of the speaker. Needless to say, it is a suggestion upon the rights or wrongs of which a medical journal offers no opinion at all.

### A MEMORIAL TO FLORENCE NIGHTINGALE.

The spontaneous desire on the part of the nation in general, and the medical and nursing profession in particular, to commemorate the services rendered by the Lady with the Lamp in a suitable manner will probably find an outlet in more than one direction. A national memorial needs careful thought and full consideration before its details can be accepted. We have no doubt that some such memorial will be raised in the near future, for Miss Nightingale's work, so self-effacingly carried out, without parade, advertisement, or agitation, runs a risk of being forgotten by the rising generation to whom she is but a name. To those that know her history and the story of her life no such memorial is necessary, for it is found in the success of her endeavours to raise the standard of hospital and private nursing in this country. Nothing can detract from the obligations under which she has laid the nation at large, and no monument of brass or marble can add to the glory that surrounds her memory or increase the reverence with which her name is regarded by those who knew her and her work. But it is, notwithstanding, desirable that a permanent tribute of respect should be paid to the great lady. The value of a memorial, remarks Hazlitt in one of his essays, is not circumscribed merely by its intrinsic, artistic, sentimental, or practical merits; it is found to a large extent in the fact that it serves to keep alive the appreciation of the worth of the person or the events commemorated. We lose reverence by degrees, and our appreciation grows lukewarm by stages. A case in point, which is specially applicable to the instance, is the memorial to Howard, the great reformer, who in some ways rendered as great a service to humanity as Miss Nightingale in other directions has done. The Bedford statue is known to few, but the Howard Society is an active association whose influence is felt in many quarters and whose power for good has been very great. Is it too much to hope that we may yet see the establishment of a Nightingale Association which will have as its chief object the furthering of the high ideals the Lady with the Lamp had before her during her eventful life? The time is opportune for the founding of such an organisation, and it is more than likely that it will have a wide scope of usefulness when established.

### THE ST. THOMAS'S SCHEME.

Meanwhile we would draw attention to the scheme Mr. Wainwright, the Treasurer to St. Thomas's Hospital, has outlined. It will be remembered that this institution is associated with the name of Miss Nightingale. "At the conclusion of the Crimean War," Mr. Wainwright writes, "the nation sought to express its gratitude by presenting to Miss Nightingale a large sum of money. This she declined to accept for her personal use, and devoted it to the establishment of a training school for nurses, and St. Thomas's Hospital was honoured by selection as the field of her work. Miss Nightingale personally undertook the organisation of the details under which the systematic training of nurses in this country was begun. The governors of St. Thomas's fully realised the importance of Miss Nightingale's scheme, and on the transference of the hospital to its present fine site they readily agreed to build a home for the accommodation of the "Nightingale School," Mrs. Wardroper, the able matron of the hospital, acting as superintendent and carrying out the founder's ideas. For over half a century this training school has continued its magnificent work, and the principles laid down at its foundation have not been altered. They have been adopted by and are the guiding spirit of innumerable training schools for nurses, not only in this country, but in every English-speaking country of the world, as well as in many foreign countries." As treasurer of St. Thomas's Hospital, Mr. Wainwright has been approached by a large number of old Nightingale nurses and others interested in nursing with the request to undertake the duty of organising a fund in her honour, such fund to serve as the "Nurses' Memorial" to Miss Nightingale. He has willingly accepted the task, and is now taking steps to form a committee as widely representative as possible of the nursing interest, for such a memorial will not be confined to Nightingale nurses, and should secure the assistance of all nurses wherever trained, and of all interested in her work. The actual form of the memorial can only be settled by the contributors themselves. A meeting will be held for the purpose of considering and deciding this important question as soon as promises or contributions have been received from a sufficient number, but, as Mr. Wainwright concludes in his letter, "there seems to be an almost unanimous feeling that the best way of honouring so dear a memory as that we treasure for our late chief is the foundation of a fund for the assistance of trained nurses."

### HOPE HOSPITAL, SALFORD.

The resignation of Dr. Hindshaw, the medical superintendent who has done such good service at the Salford Poor-Law Infirmary, has caused the Guardians to advertise the vacancy and to invite applications from qualified practitioners who have had previous administrative experience in Poor Law or general infirmary work. It was essential to the welfare of the patients and the efficiency of the Hope Hospital that the Guardians should strictly adhere to the terms of their advertisement and disqualify all candidates who did not possess the administrative experience in question. With an ineptitude which has made this Board of Guardians notorious, the majority, however, appointed a gentleman who does not appear to have had the advertised qualifications, and the President of the Local Government Board, with that splendid watchfulness over the best interests of the poor sufferers for which his department is so largely responsible, at once communicated with the Hope Board, asking the Guardians to supply a statement of what previous administrative experience the selected candidate had had which qualified him to undertake the onerous duties of medical superintendent. Instead of promptly retracing the false step which the majority of the Guardians had taken, the *Manchester Evening News* reports that the majority moved and carried an amendment to the resolution moved by Mrs. Shutt to comply with the request of the Local Government Board, and reaffirmed their previous decision to ignore the conditions of their own advertisement. We have every confidence that the President of the Local Government Board will stick to his guns and insist that the new medical superintendent of the Hope Hospital, whoever he may be, shall at least possess the best qualifications and the largest previous administrative experience in a Poor Law or general infirmary which it is possible to secure.

### THIS WEEK'S DRUG MARKET.

Among the alterations in the price of drugs which have taken place during the week is a further reduction of 3d. per oz. in the price of codeine. The opium market in London is very quiet and the position practically unchanged. Both tartaric acid and cream of tartar are dearer. A fair business has been done in cascara sagrada at higher prices, and the general belief is that prices will further advance. Camomiles, American peppermint oil, and oil of eucalyptus are also tending dearer.

